# A third (booster) dose of the inactivated SARS-CoV-2 vaccine elicits immunogenicity and T follicular helper cell responses in people living with HIV

**DOI:** 10.3389/fimmu.2023.1264160

**Published:** 2023-11-15

**Authors:** Zhengchao Lv, Songqin Lv, Qin Li, Yafei Xia, Zaineng Feng, Haohong Zhang, Haihao Yang, Zhao Wu, Nanting Zou, Qingyan Mo, Qianlan Gu, Sai Ying, Xicheng Wang, Dongdong Qin, Chunping Wan

**Affiliations:** ^1^ School of Clinical Medicine, School of Pharmacy and School of Basic Medicine, Yunnan University of Chinese Medicine, Kunming, China; ^2^ Department of AIDS Clinical Treatment, Yunnan Provincial Hospital for Infectious Diseases, Kunming, China; ^3^ Medical Laboratory, The Third People’s Hospital of Kunming, Kunming, China; ^4^ Infectious Disease Department, The First People’s Hospital of Xuan Wei, Qujing, China; ^5^ Infectious Disease Department, Malipo Country People’s Hospital, Wenshan, China; ^6^ Pharmacy Department, Zhengxiong Country Hospital of Traditional Medicine, Zhaotong, China; ^7^ Key Laboratory of Traditional Chinese Medicine for Prevention and Treatment of Neuropsychiatric Diseases, Yunnan University of Chinese Medicine, Kunming, China

**Keywords:** booster dose, inactivated SARS-CoV-2 vaccines, immunogenicity, T follicular helper cells, PLWH

## Abstract

**Introduction:**

This study sought to explore the immunogenicity of a booster dose of an inactivated severe acute respiratory syndrome coronavirus 2 (SARS-CoV-2) vaccine in people living with human immunodeficiency virus (HIV) and identify the factors affecting the magnitude of anti-SARS-CoV-2 antibody levels.

**Materials and methods:**

A total of 34 people living with HIV (PLWH) and 34 healthy donors (HD) were administered a booster dose of the same SARS-CoV-2 vaccine. Anti-SARS-CoV-2 antibody and immunoglobulin G (IgG) levels were measured using the SARS-CoV-2 S protein neutralizing antibody Enzyme-Linked Immunosorbent Assay (ELISA) and 2019-nCov IgG Chemiluminescent Immunoassay Microparticles, respectively. Spearman correlation analysis was used to measure the correlation between laboratory markers and neutralizing antibody and IgG levels. Peripheral blood mononuclear cells (PBMCs) were extracted from each subject using density gradient centrifugation and the numbers of memory T and T follicular helper (Tfh) cells were determined using flow cytometry.

**Results:**

PLWH had a marked reduction in CD4 and B cell levels that was accompanied by a lower CD4/CD8 T cell ratio. However, those who received a supplementary dose of inactivated SARS-CoV-2 vaccines exhibited antibody positivity rates that were analogous to levels previously observed. The booster vaccine led to a reduction in IgG and neutralizing antibody levels and the amplitude of this decline was substantially higher in the PLWH than HD group. Correlation analyses revealed a strong correlation between neutralizing antibody levels and the count and proportion of CD4 cells. Anti-SARS-CoV-2 IgG antibody levels followed a similar trend. The expression of memory T and Tfh cells was considerably lower in the PLWH than in the HD group.

**Discussion:**

PLWH had an attenuated immune response to a third (booster) administration of an inactivated SARS-CoV-2 vaccine, as shown by lower neutralizing antibody and IgG levels. This could be attributed to the reduced responsiveness of CD4 cells, particularly memory T and cTfh subsets. CD4 and cTfh cells may serve as pivotal markers of enduring and protective antibody levels. Vaccination dose recalibration may be critical for HIV-positive individuals, particularly those with a lower proportion of CD4 and Tfh cells.

## 1 Introduction

Ongoing changes in the genome of the severe acute respiratory syndrome coronavirus 2 (SARS-CoV-2) and alterations in the S protein have allowed SARS-CoV-2 infection to persist as a global pandemic (1, 2). According to a report by the World Health Organization on July 20, 2023, approximately 7 million SARS-CoV-2 fatalities have occurred worldwide. During the early phase of the pandemic, when vaccination was not yet available, the correct use of protective equipment and early identification were essential to avoiding nosocomial clusters (3). The development of anti-COVID-19 vaccines has been essential to managing infection in both clinical trials and real-life scenarios (4, 5). A rapid living systematic evidence synthesis and meta-analysis illustrated that vaccine effectiveness has generally decreased over time (6). Neutralizing antibody levels induced by inactivated SARS-CoV-2 vaccines were shown to persist for only 6 months following the administration of two doses (7). Thus, a third dose of vaccine was recommended. Recent data suggests that the efficacy of CoronaVac^®^ in combating COVID-19 rose from 56% to 80% 14 days following the administration of a booster dose (8). Three vaccine doses are also shown to induce a stronger neutralizing antibody response than two doses of the inactivated SARS-CoV-2 vaccine (9).

Our previous research found that people living with HIV (PLWH) and healthy donors (HD) had similar rates of positive neutralizing antibodies. However, PLWH exhibited less robust reactions to inactivated SARS-CoV-2 vaccines than their healthy counterparts. In addition, a significant correlation between neutralizing antibodies and CD4 and B cell levels in PLWH indicated the necessity of an additional dose for individuals infected with HIV (10). However, until now, the impact of an additional vaccine dose on the immune response has been dependent on the overall population or vaccines based on messenger RNA (mRNA) and adenovirus vectors. The immune response to an additional dose of the deactivated SARS-CoV-2 vaccine in PWLH remains largely unknown (11). The current study sought to expand on our prior findings by measuring neutralizing antibody, immunoglobulin G (IgG), and specific follicular T helper (Tfh) cell levels in PLWH who received a third (booster) dose of the inactivated SARS-CoV-2 vaccine. The findings should offer valuable information on the immune reaction to a supplementary vaccine dose in PWLH.

## 2 Materials and methods

### 2.1 Study design and participants

#### 2.1.1 Study design

This study was conducted in three cities of Yunnan province (Xuanwei, Malipo, and Kunming) between December 2021 and March 2022. All participants, including PLWH and HD, received a third (booster) dose of an inactivated SARS-CoV-2 vaccine (CoronaVac or BBIBP-CorV).

#### 2.1.2 Participants

PLWH who were 18–59 years of age, were willing to participate in the study, including survey completion, blood sample collection, and relevant laboratory testing, received a third (booster) dose of an inactivated SARS-CoV-2 vaccine (CoronaVac or BBIBP-CorV), and were diagnosed with HIV, were included in the study. Individuals with severe hearing loss, impaired vision, or intellectual disability observed by the interviewers, no history of exposure to COVID-19, or a major psychiatric illness were excluded. The experimenter told all PLWH enrollees about the study purpose and procedures. Identifiable information was kept confidential, and all participants provided written informed consent before screening for eligibility. A total of 34 HIV-positive participants were also enrolled from Xuanwei City People’s Hospital and Malipo Country People’s Hospital. A control group of 34 healthy (blood) donors was enrolled from a neighborhood in Kunming City, Yunnan Province, China. Participants were excluded if they had no prior contact with COVID-19 or tested positive for SARS-CoV-2 through pharyngeal or anal swab tests prior to enrollment. The Ethics Committee of Yunnan Provincial Traditional Chinese Medicine Hospital approved this experimental protocol (NO. K [2021]015). All participants provided written informed consent forms, and all procedures were carried out in compliance with the guidelines set by the International Conference on Harmonization.

### 2.2 Lymphocyte quantification

The BD Multitest™ reagent (BD Pharmingen) was used to calculate the absolute T and B cell counts and percentages, and the levels of CD4 and CD8 T cell subpopulations as previously described (10). The specimens were obtained using a flow cytometer (FACSCanto™ II; BD Biosciences).

### 2.3 Anti-SARS-CoV-2 neutralization antibody levels

Anti-SARS-CoV-2 neutralization antibody levels were measured by Enzyme-Linked Immunosorbent Assay (ELISA) (SN: EKnCo v001, Frdbio, Wuhan, China) as previously described (10). In brief, serum was obtained from individuals 18 days after the third vaccine dose. Serum samples (50 µL) were added into wells that were pre-coated with Human ACE2 protein, and incubated with HRP- SARS-CoV-2 Spike RBD at 37°C for 1 h. The plate was washed three times and 50 µL of the substrates were added to each well and incubated at 3°C for 20 min. Stop solution was added to terminate the reaction and the plates were read at OD 450 nm. Neutralizing antibodies in the samples competed with ACE2 to bind to HRP-SARS-CoV-2 Spike RBD. The following percentage inhibition formula was used:


Percentage of inhibition=(1−(OD 450 of sample/OD 450 of negative control))×100%

A threshold of 20% was used to delineate negative and positive outcomes.

### 2.4 Anti-SARS-CoV-2 IgG antibody levels

The 2019-nCov IgG Chemiluminescent Immunoassay Microparticles (30041613CM01, Autobio, Zhengzhou, China) were used to measure anti-SARS-CoV-2 IgG antibody levels according to the manufacturer’s instructions. The serum was collected from participants approximately 18 days following administration of the third vaccine dose. After incubation, the samples were rinsed three times with a washing solution and then introduced to a chromogenic substrate. The luminous intensity was detected using a Luminoskan Ascent.

### 2.5 Flow cytometry

Approximately 18 days after the third vaccine injection, peripheral blood samples were collected to quantify memory T cells (CD3+CD4+CD45RO+ memory T cells) and T follicular helper cells (CD4+CXCR5+PD1+Tfh). PBMCs were obtained by centrifuging peripheral blood using a density gradient, suspended in a freezing medium, and stored for use in future experiments. The cryopreserved PBMCs were defrosted in a water bath at 37°C, washed, and suspended in an RPMI 1640 solution containing 10% fetal bovine serum. To measure memory T cells, the PBMCs (5×10^5^ cells/tube) were blocked with mouse anti-human CD16/CD32 antibody (2.4G2, BD Pharmingen) and then fluorescently labeled at 4°C for 15 minutes with FITC-conjugated-anti-human CD3 (HIT3a, Biolegend), PE-Cy7-conjugated anti-human CD4 (RPA-T4, Biolegend), or APC-conjugated anti-human CD45RO (UCHL1, Biolegend). To measure Tfh cells, PBMCs (1×10^6^ cells/tube) were blocked with mouse anti-human CD16/CD32 antibody and then fluorescently labeled with PE-Cy7-conjugated anti-human programmed cell death 1 (PD-1) (EH12.1, Biolegend), V500-conjugated anti-human CD4 (RPA-T4, BD Horizon™), or Pacific Blue™-conjugated anti-human-C-X-C motif chemokine receptor 5 (CXCR5) (J252D4, Biolegend). The specimens were obtained with a flow cytometer (FACSCanto™ II; BD Biosciences) and analyzed using FlowJo software (Tree Star).

### 2.6 Statistical analysis

SPSS 20.0 was used to conduct the analysis and the Kolmogorov-Smirnov test was used to examine if a variable adhered to a Gaussian distribution. The data that followed a normal distribution was presented as the mean ± SEM (standard error of the mean). An independent sample t-test was used to compare PLWH and HD. The median (interquartile range) was used to represent data that did not have a normal distribution, and a non-parametric test was used to compare the two groups. The enumeration data was compared using a χ^2^ test and the correlation was assessed by Spearman analysis. Statistical significance was determined as p<0.05.

## 3 Results

### 3.1 HIV-positive patient characteristics and vaccine safety

The demographics of the study groups are summarized in **Table 1**. Sixty-eight participants were included in the study, all of whom received a third injection of one of the inactivated SARS-CoV-2 vaccines (CoronaVac or BBIBP-CorV). This included one group of 34 individuals who were in good health and tested negative for HIV (HD group) and a second group of 34 PLWH. While 18 (52.94%) participants in the HD group were male, 17 participants (47.75%) in the PLWH group were male. The interval between the third vaccination and serum sampling was matched in both groups [PLWH versus HD: 17.50 days (IQR 15.75–21.00) vs 18.00 days (IQR 16.75–25.00), P=0.151] (**Table 1**). While no significant difference in gender and interval time was observed between the HD and PLWH groups (P >0.05), they were not matched by age [PLWH versus HD: 43.50 years (IQR 37.75–47.25) vs 30.00 years (IQR 22.75–39.00), P<0.001] (**Table 1**). However, our previous study and other reports have demonstrated no significant associations between age and neutralizing antibody levels (10, 12). The average age of HIV diagnosis was 9 years and the viral load was undetectable in 33 of 34 individuals (97.00%). All HIV-positive participants received daily antiretroviral therapy (ART) and 17 of 34 (50.00%) had CD4+ T cell counts<500.

**Table 1:** Demographic characteristics of the PLWH and HD groups.

Variable	HD(n=34)	PLWH(n=34)	Z/χ^2^	P
**Age, [Median(Q3-Q1), years]**	30.00 (22.75, 39.00)	43.50 (37.75, 47.25)	-4.438	<0.001
Gender				
**Male, n (%)**	18 (52.94%)	17 (50.00%)	0.059	0.808
**Female, n (%)**	16 (47.06%)	17 (50.00%)		
**Interval Time [Median (Q3-Q1), days]**	18.00 (16.75, 25.00)	17.50 (15.75, 21.00)	-1.436	0.151
Characteristics related to HIV infection				
**Time since HIV diagnosis, years [Median (Q3-Q1)]**	N.A	9 (5, 10)	N.A	N.A.
Viral load (cp/mL), n (%)				
**Undetectable (≤60)**	N.A	33 (97.06%)	N.A	N.A.
**61-200**	N.A	1 (2.94%)	N.A	N.A.
CD4^+^T-cell counts (cells/μL)				
**<500, n (%)**	N.A	17 (50.00%)	N.A	N.A.
**500 - 1000, n (%)**	N.A	16 (47.06%)	N.A	N.A.
**>1000, n (%)**	N.A	1 (2.94%)	N.A	N.A.
ART regimens				
**TDF + 3TC + EFV**	N.A	7 (20.59%)	N.A	N.A.
**TDF + 3TC + LPV/r**	N.A	4 (11.76%)	N.A	N.A.
**AZT + 3TC + LPV/r**	N.A	7 (20.59%)	N.A	N.A.
**AZT + 3TC +NVP**	N.A	7 (20.59%)	N.A	N.A.
**AZT + 3TC + EFV**	N.A	6 (17.65%)	N.A	N.A.
**Others**	N.A	3 (8.82%)	N.A	N.A.

P values were obtained using the χ2 test. N.A,: not applicable. ART, antiretroviral therapy.

Self-reported local and systematic SARS-CoV-2 vaccine-related adverse events are listed in **Table 2**. A third dose of the inactivated SARS-CoV-2 vaccine was relatively safe for HIV-positive individuals, with pain at the vaccination site being the most common adverse reaction. Interestingly, the PLWH group had a lower proportion of local adverse events (P<0.01) but a similar level of systematic adverse events (P >0.05) than the HD group. It is possible that the pain threshold of the HIV-positive participants was higher than the HDs because HIV-positive individuals are accustomed to having blood drawn to detect their immune status during ART treatment.

**Table 2:** Self-reported SARS-CoV-2 vaccine-related local and systematic adverse events in the PLWH and HD groups.

Variable	HD(n=34)	PLWH(n=34)	P
Local adverse events, n (%)			
**None**	21 (61.76)	31 (91.18)	
**Pain at the injection point**	8 (23.53)	2 (5.88)	
**Pain, redness and swelling at the injection site**	2 (5.88)	0 (0.00)	
**Pain at the injection point, and muscle pain**	2 (5.88)	0 (0.00)	
**Oropharyngeal pain**	1 (2.94)	0 (0.00)	
**Muscle pain, joint pain**	0 (0.00)	1 (2.94)	
**Any of above**	13 (38.24)	3 (8.82)	0.004
Systematic adverse events, n (%)			
**None**	29 (85.29)	32 (94.12)	
**Disgusting**	2 (5.88)	0 (0.00)	
**Nausea, vomiting**	1 (2.94)	0 (0.00)	
**Fatigue**	2 (5.88)	2 (5.88)	
**Any of above**	5 (14.71)	2 (5.88)	0.231

P values were obtained using the χ2 test.

### 3.2 Lymphocyte quantification

**Figure 1:**
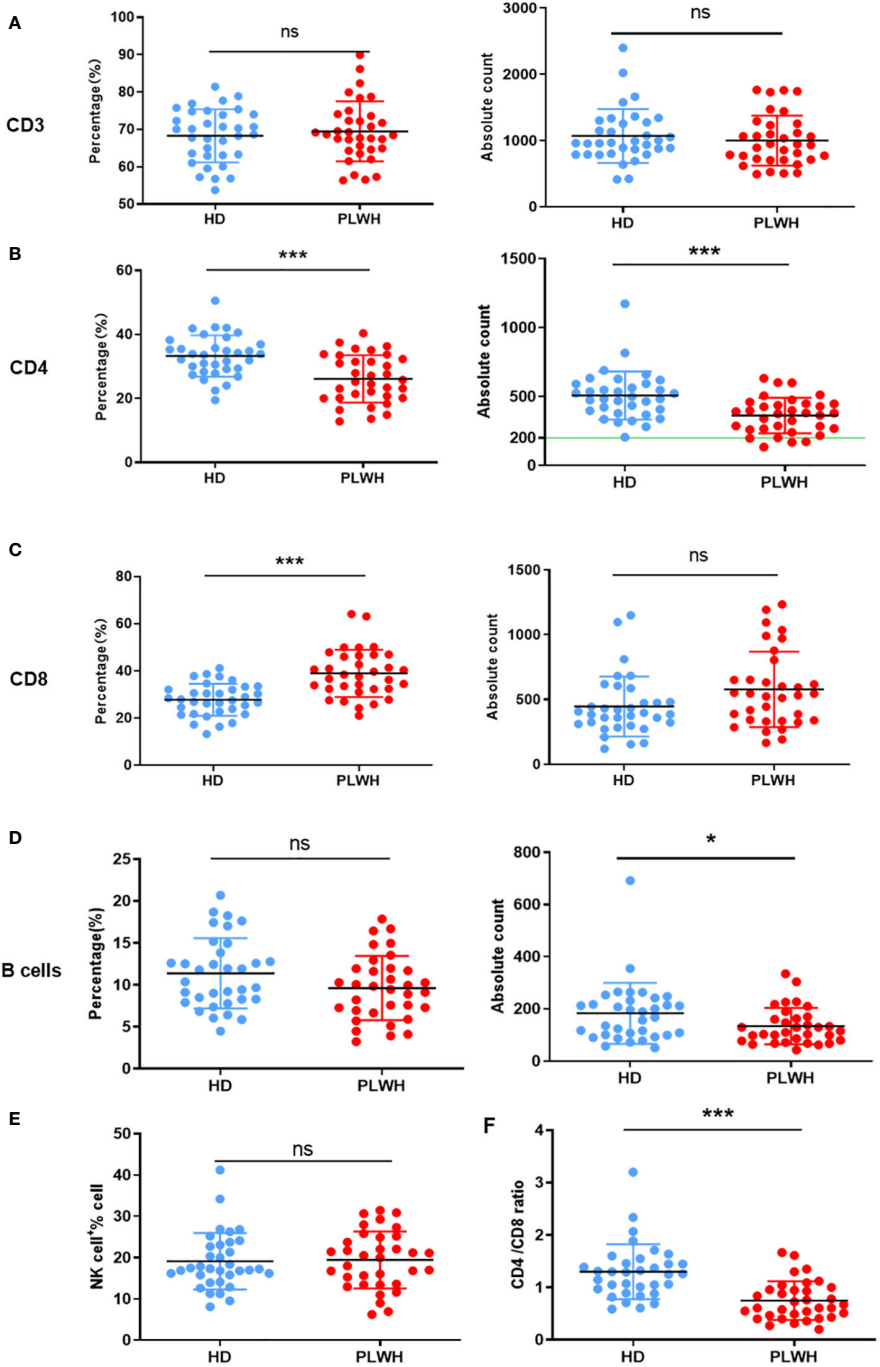
Quantification of lymphocytes in peripheral whole blood. Peripheral whole blood samples taken about 18 days after the third (booster) injection of the inactivated SARS-CoV-2 vaccine were collected and stained with the 6-Color TBNK reagent. The erythrocytes were then lysed using BD FACS™ Lysing Solution and the samples were obtained using a flow cytometer. CD3 **(A)**, CD4 **(B)**, CD8 **(C)**, B cells **(D)** and NK cells **(E)** expressions were analyzed, and the CD4/CD8 ratio **(F)** was calculated. ns: not significant, *p<0.05, ***p<0.001.

The percentage and absolute counts of CD4 and B cells were considerably lower in the PLWH group than in the HD group after a booster dose of the inactivated vaccine (P<0.001 and P<0.05, respectively). The percentage of CD8 cells was significantly higher in the PLWH group than in the HD group (P<0.001). Thus, the ratio of CD4/CD8 cells was also considerably lower in the PLWH group (P<0.001). These findings are consistent with the pathogenic characteristics of HIV-positive individuals (**Figure 1**).

### 3.3 Neutralizing and IgG antibody correlation analysis

**Figure 2:**
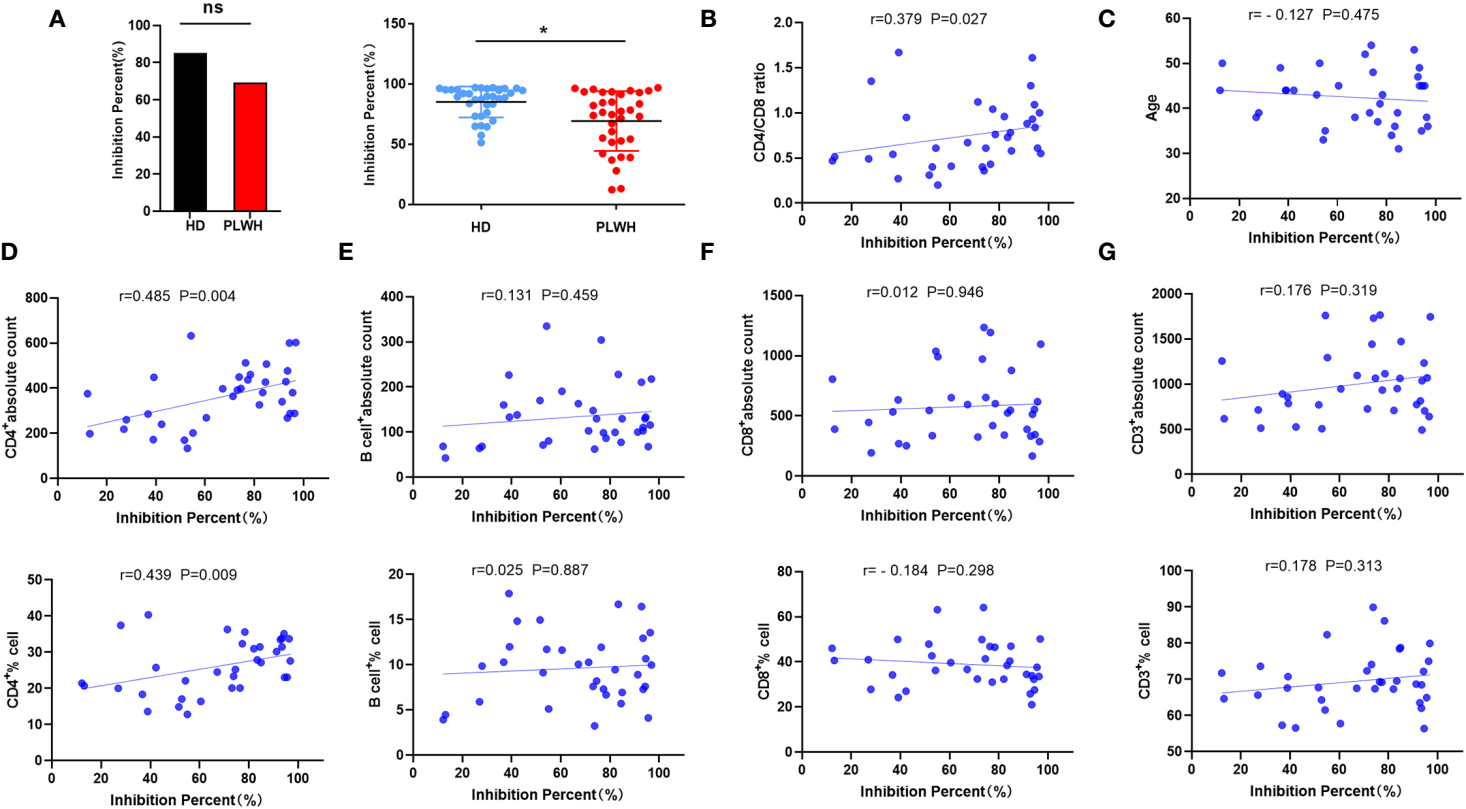
Neutralizing antibody measurement and correlation analysis. Neutralizing antibody levels were measured 18 days after administration of a booster dose of the inactivated SARS-CoV-2 vaccine using a competitive ELISA **(A)**. Spearman or Pearson correlation analysis was used to assess the correlation between laboratory indicators and neutralizing antibody levels in the PLWH group **(B-G)**. ns: not significant, *p<0.05.

Anti-spike protein antibodies are likely to be effective against SARS-CoV-2 (13). Approximately 18 days after receipt of the booster shot, IgG and neutralizing antibodies targeting the spike RBD of SARS-CoV-2 were identified. The seropositivity rate in the HD and PLWH groups was 100% and 94.11% (32 of 34), respectively (**Figure 2**). Neutralizing antibody seropositivity rates were significantly lower in the HD than in the PLWH groups (P<0.05) (**Figure 2A**). These findings align with our previous reports on the immunogenicity of PLWH who received a second injection and suggest that a booster dose of the vaccine elicited a lower immune response in HIV-positive individuals than in the general population. The results showed a positive correlation between neutralization antibody levels and the CD4/CD8 ratio (**Figure 2B**). No significant correlation was observed between age and neutralization antibody levels (**Figure 2C**). The CD4 cell count and CD4 percentage were positively correlated with neutralization antibody levels (**Figure 2D**). While, no significant correlations were observed between the B cell count and percentage (**Figure 2E**), CD8 cell count and percentage (**Figure 2F**), as well as CD3 cell count and percentage (**Figure 2G**), and neutralizing antibody levels.

**Figure 3:**
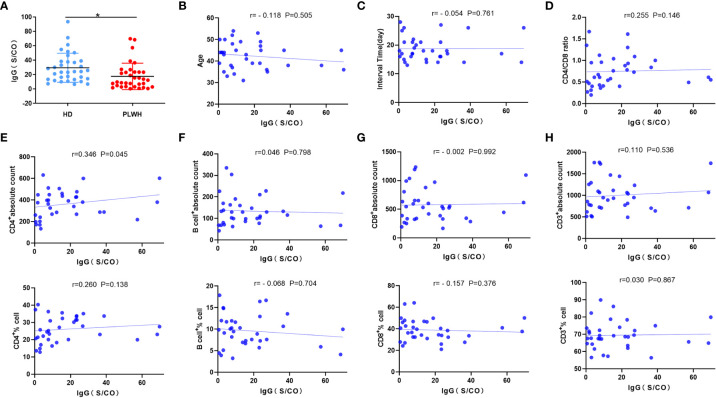
Anti-SARS-CoV-2 IgG antibody measurement and correlation analysis. Serum samples were obtained from participants about 18 days after the third injection of the vaccine, and anti-SARS-CoV-2 IgG antibodies were measured using 2019-nCovIgG Chemiluminescent Immunoassay Microparticles **(A)**. Spearman or Pearson correlation analysis was used to assess the correlation between laboratory indicators and neutralizing antibody levels in the PLWH group **(B–H)**. *p<0.05.

Mean anti-RBD IgG responses were significantly lower in the PLWH group than in the HD group (P<0.05) (**Figure 3A**). While there was a positive correlation between the IgG antibody level and CD4 count (P<0.05) (**Figure 3E**), there was no correlation between other laboratory indicators and the anti-SARS-CoV-2 IgG antibody level (P >0.05) (**Figures 3B–D, F–H**). These findings indicated that CD4 cells play an important role in the production of anti-SARS-CoV-2 IgG antibodies.

### 3.4 Memory T cells and T follicular helper cells

**Figure 4:**
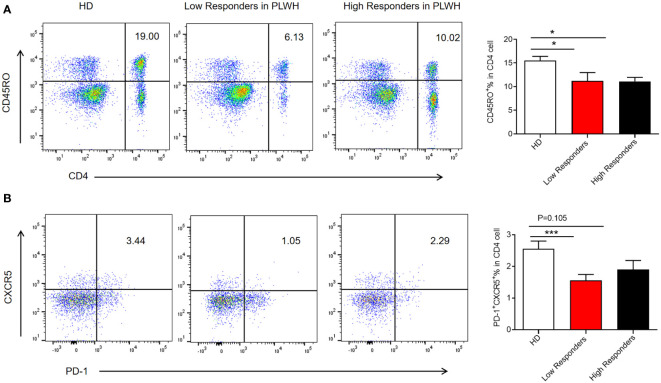
Expression of memory T cells and follicular helper T cells in low and high responders. PBMCs were isolated from participants using density gradient centrifugation, resuspended in a cell freezing medium, and stored in liquid nitrogen. The frozen PBMCs were thawed in a 37°C water bath and blocked with mouse anti-human CD16/CD32. The cells were then stained with the appropriate antibodies. Samples were acquired using a flow cytometer and analyzed using FlowJo software **(A, B)**. The results were presented as the mean ± SEM. N = 10, *p<0.05, ***p<0.001 vs. the HD group.

Memory T cells and Tfh cells, particularly CD4+ T cells, help to coordinate immunological reactions and promote the production of antibodies. These results demonstrate that immunogenicity was lower in the PLWH than in the HD group. To further explore the immune mechanism, the expression of memory T and Tfh cells was assessed in low responders (inhibition level<45%) and high responders (inhibition level >80%) to inactivated SARS-CoV-2 vaccines in the PLWH group using flow cytometry. The proportion of memory T cells was considerably lower in the low responder PLWH group than in the HD group (P<0.05) (**Figure 4A**). Low responders also had decreased expression of Tfh (CD4+ CXCR5^+^PD1^+^Tfh) than individuals in the HD group (P<0.001) (**Figure 4B**). However, high responders in the PLWH group showed no difference in Tfh cell expression (**Figure 4B**). In summary, the reduced reaction to the inactivated SARS-CoV-2 vaccine in low responders correlated with lower expression of Tfh cells.

## 4 Discussion

Vaccination remains the most effective way to reduce the effects of COVID-19 (14). Neutralization antibodies restrict the entry of SARS-CoV-2 into host cells by preventing spikes from interacting with ACE2 (15), thereby exerting neutralizing and antiviral activity by eliminating free virions or infected cells through Fc effector mechanisms (16, 17). Neutralizing monoclonal antibodies that are passively transferred into animal models have protective benefits against experimental SARS-CoV-2 infection (18) and neutralizing antibody titers correlate positively with vaccine efficacy (19). HIV infection leads to chronic inflammation and immune activation, aging the immune system and reducing its responsiveness. Many HIV-positive patients experience persistent T cell activation and exhaustion for years, suggesting that they may have an impaired SARS-CoV-2 elicited specific immune response (20). During the first period of COVID-19, most data on vaccine safety and effectiveness were obtained from the general population, with very little information on the immunogenicity of inactivated SARS-CoV-2 vaccines in HIV-positive individuals. Huang et al. found that PLWH had lower neutralizing antibody, total antibody, and S-IgG levels and T-cell-specific immune responses, than HIV-negative individuals. PLWH also had reduced immunogenicity to the inactivated SARS-CoV-2 vaccines (21). However, Frater et al. found no difference in the magnitude or persistence of SARS-CoV-2 spike-specific humoral or cellular responses in HIV-positive individuals, supporting that ChAdOx1 nCoV-19 was safe and immunogenic in this population (12). In contrast, our previous research found that PLWH had less robust reactions to inactivated SARS-CoV-2 vaccines. A significant correlation was shown between neutralizing antibody levels and CD4 and B cell levels in PLWH. Thus, controversy remains about the efficacy, safety, and tolerance of SARS-CoV-2 vaccines in PLWH.

While two vaccine doses induce substantial neutralizing antibody titers 2–4 weeks after the second dose, titers decline 6–8 months after injection. Thus, the Chinese government sought to investigate the efficacy of a booster dose on the general population. The immunological response to a third (booster) dose of the vaccine in PLWH, however, remains poorly understood. HIV-infected individuals, particularly those with lower CD4 T and B cell counts, may require a booster dose or dose modification (10). To address this, the current study assessed the immunological reaction generated by an additional dose of the deactivated SARS-CoV-2 vaccine to PLWH. While similar levels of positive neutralizing antibodies were found in both the PLWH and HD groups, the percentage of inhibition was significantly lower in the PLWH group. Cecilia et al. similarly found that vaccine-induced IgG levels were similar between the PLWH and control groups. However, a slightly lower proportion of PLWH maintained vaccine-induced anti-S IgG immunity 6 months after the second dose (22). The vaccine was very well tolerated in both groups after the booster dose (23). In the current study, a strong correlation was found between neutralizing antibody levels and the CD4 cell count, CD4 cell proportion, and CD4/CD8 ratio. CD4 T cells contribute to viral clearance by promoting the generation of CD8 T cells, mediating direct cytolytic activity, and secreting anti-viral cytokines (24). A low CD4/CD8 ratio is a clinically available biomarker of immune activation and correlates with a high risk of mortality in ART-treated HIV-infected patients (25). In SIV-infected macaques, decreased antibody and cell mediated immune (CMI) responses are associated with reduced circulating Tfh counts and aberrant CD4/CD8 ratios, respectively. This indicates that SIV-mediated dysregulation of CD4 T cells impairs vaccine-specific immunity, inducing immune senescence and disruption of CMI responses. Immune reconstitution and the normalization of CD4/CD8 ratios by ART may be beneficial for PLWH (26).

Naïve CD4 T cell activation can induce their differentiation into distinct subsets of effector Tfh cells with varied functions. Tfh cells are a specialized subgroup of CD4 T cells that provide critical “help” to B cells, controlling the germinal center (GC) reaction, which induces the production of high-affinity antibodies and memory B cells (27). Human Tfh cells in lymphoid tissues, characterized by a lack of CC chemokine receptor type 7, correlate with expression of the chemokine receptors, CXCR5 and PD-1 (28, 29). The Tfh cell-specific transcription factor is B cell lymphoma 6 in lymphoid tissues (30). To date, assessment of Tfh cells in humans has been prevented by the challenge of acquiring secondary lymphoid tissue. Thus, circulating Tfh (cTfh, characterized as CXCR5^+^PD1^+^CD4 T cells) perform a vital role in defining Tfh responses in GC (31, 32). During SARS-CoV-2 infection and vaccination-induced immune responses, cTFH responses can provide critical insight into the magnitude and qualitative aspects of the humoral immune response. Mudd et al. found that the SARS-CoV-2 mRNA vaccine elicits an S-specific TFH cell response that plays a key role in establishing long-term immunity (33). However, it has remained clear whether cTfh responses are induced by inactivated SARS-CoV-2 vaccines in PLWH. To address this, we measured cTfh cell expression among weak/non-responders in the HIV-positive group. Interestingly, the proportion of cTfh cells was significantly lower in weak/non-responders than in individuals in the HD group. IL-21 is an essential cytokine produced by Tfh cells that plays a major role in mediating the GC reaction (34, 35). These findings indicate that cTfh responses may be used to monitor treatments that induce the production of durable and protective neutralizing antibody responses. Given the small size of the weak/non-responder group, however, more research is needed to confirm our findings.

The current study had several limitations. First, while some laboratory indicators (cTfh and CD4) were dramatically lower in the PLWH group than in the HD group, it remains unknown what role they played in the generation of antibodies. Second, this was an open-label study without randomization so it was challenging to recruit individuals of comparable age for each group. However, the cohorts were similarly matched by gender and the interval between sampling and vaccination. Prior studies found no robust correlation between age and neutralizing antibodies among PLWH. Third, the patient sample was relatively small and will require confirmation using a large sample study.

In conclusion, our study indicated that administering an additional dose of any of the inactive SARS-CoV-2 vaccines induced a similar level of effective neutralizing antibodies in PLWH and healthy individuals. However, PLWH had diminished reactions to the vaccine, as evidenced by lower levels of neutralizing antibodies, IgG, CD4 cells, memory T cells, and Tfh cells. These indicators could be used to assess the development of an effective immune response against SARS-CoV-2.

## Data availability statement

The raw data supporting the conclusions of this article will be made available by the authors, without undue reservation.

## Ethics statement

The studies involving humans were approved by the institutional review board of the Yunnan Provincial Traditional Medicine Hospital Ethics Committee (NO. K [2021]015). The studies were conducted in accordance with the local legislation and institutional requirements. The participants provided their written informed consent to participate in this study.

## Author contributions

ZL: Writing – original draft. SL: Writing – original draft. QL: Data curation, Software, Writing – review & editing. YX: Conceptualization, Data curation, Writing – review & editing. ZF: Data curation, Methodology, Writing – review & editing. HZ: Data curation, Formal Analysis, Methodology, Writing – review & editing. HY: Conceptualization, Methodology, Software, Writing – review & editing. ZW: Data curation, Methodology, Writing – review & editing. NZ: Data curation, Methodology, Writing – review & editing. QM: Data curation, Methodology, Writing – review & editing. QG: Software, Supervision, Writing – review & editing. SY: Data curation, Formal Analysis, Investigation, Methodology, Software, Writing – review & editing. XW: Conceptualization, Investigation, Software, Supervision, Validation, Writing – review & editing. DQ: Conceptualization, Data curation, Investigation, Supervision, Writing – original draft, Writing – review & editing. CW: Conceptualization, Data curation, Formal Analysis, Investigation, Methodology, Supervision, Writing – original draft, Writing – review & editing.
